# Colonic levels of vasoactive intestinal peptide decrease during infection and exogenous VIP protects epithelial mitochondria against the negative effects of IFNγ and TNFα induced during *Citrobacter rodentium* infection

**DOI:** 10.1371/journal.pone.0204567

**Published:** 2018-09-25

**Authors:** Arpan K. Maiti, Sinan Sharba, Nazanin Navabi, Sara K. Lindén

**Affiliations:** Department of Medical Biochemistry and Cell Biology, Sahlgrenska Academy, University of Gothenburg, Gothenburg, Sweden; University of Kansas, UNITED STATES

## Abstract

*Citrobacter rodentium* infection is a model for infection with attaching and effacing pathogens, such as enteropathogenic *Escherichia coli*. The vasoactive intestinal peptide (VIP) has emerged as an anti-inflammatory agent, documented to inhibit Th1 immune responses and successfully treat animal models of inflammation. VIP is also a mucus secretagogue. Here, we found that colonic levels of VIP decrease during murine *C*. *rodentium* infection with a similar time dependency as measurements reflecting mitochondrial function and epithelial integrity. The decrease in VIP appears mainly driven by changes in the cytokine environment, as no changes in VIP levels were detected in infected mice lacking interferon gamma (IFNγ). VIP supplementation alleviated the reduction of activity and levels of mitochondrial respiratory complexes I and IV, mitochondrial phosphorylation capacity, transmembrane potential and ATP generation caused by IFNγ, TNFα and *C*. *rodentium* infection, in an *in vitro* mucosal surface. Similarly, VIP treatment regimens that included the day 5–10 post infection period alleviated decreases in enzyme complexes I and IV, phosphorylation capacity, mitochondrial transmembrane potential and ATP generation as well as increased apoptosis levels during murine infection with *C*. *rodentium*. However, VIP treatment failed to alleviate colitis, although there was a tendency to decreased pathogen density in contact with the epithelium and in the spleen. Both *in vivo* and *in vitro*, NO generation increased during *C*. *rodentium* infection, which was alleviated by VIP. Thus, therapeutic VIP administration to restore the decreased levels during infection had beneficial effects on epithelial cells and their mitochondria, but not on the overall infection outcome.

## Introduction

Murine *Citrobacter rodentium* infection models infection with human enterohemorrhagic (EHEC) and enteropathogenic *Escherichia coli* (EPEC) [1–3]. Infection induces colitis with a mild transmural inflammatory infiltrate, colonic epithelial damage, goblet cell depletion and colonic crypt hyperplasia [4–6]. Concomitantly, colonic epithelial cell death is also observed, both at the luminal surface and base of the crypts [1, 7–9]. The attaching and effacing (A/E) pathogen *C*. *rodentium* mainly infects the luminal epithelial cells through the formation of a type III secretion system (T3SS), which initiates the host cell death pathway by inducing effector molecules like EspF and Map that translocate into the host mitochondria disrupting mitochondrial function [10–13]. In addition, enhanced levels of pro-inflammatory cytokines induce mitochondrial dysfunction [14], which contributes to colonic epithelial cell death during infection. From the observed Th1 response, mainly upregulation of interferon gamma (IFNγ) and tumor necrosis factor alpha (TNFα) occurring during the mid and late infection phases, led to severe mitochondrial dysfunction involving loss of complex-I and IV quantity and activity accompanied by a reduction in mitochondrial phosphorylation capacity, membrane potential and mitochondrial ATP generation [14].

In recent years, the vasoactive intestinal peptide (VIP) has emerged as a potent anti-inflammatory agent with the ability to affect multiple organs, including the respiratory, cardiovascular and gastrointestinal systems [15]. VIP has been documented to inhibit Th1 immune responses, mainly by downregulating production of TNF-α, IFN-γ and IL-12 [16–18], and successfully treat animal models of inflammation including endotoxic shock [19], rheumatoid arthritis [20] and delayed-type hypersensitivity [21]. Under normal physiological conditions, this neuropeptide is found widely distributed in the peripheral and central neurons and is expressed in the colon. The VIPergic neurons directly innervate intestinal epithelial crypt cells regulating intestinal ion and fluid secretion [22, 23] and epithelial barrier homeostasis [24]. Reports on VIP expression during animal [25–27] and human [28–32] intestinal inflammation range from no alteration in the submucosal and myenteric plexuses [33], increase in the mucosal neuronal VIP density [34] and reduction in immunoreactivity in the mucosa [27, 35].

Several studies have highlighted VIP as a potential agent for treating clinical and histopathological severity of colitis [30,31]. In the *C*. *rodentium* induced colitis model prophylactic administration of VIP ameliorated the colitis-induced epithelial damage compared with non-treated controls, though only a single time point at mid infection was investigated [36]. In the murine TNBS induced colitis model, one study found that VIP reduced the clinical and histopathologic features of colitis, whereas another study did not [37,38]. Furthermore, a few reports exist that indicate a beneficial impact of VIP in maintaining morphological integrity and function of mitochondria in non-intestinal tissues [36, 39]. However, the impact of VIP on mitochondrial dysfunction in colonic epithelial cells during C *rodentium* infection remains uninvestigated.

Here we investigate the effects of VIP on the mitochondrial dysfunction during *in vivo* and *in vitro C*. *rodentium* infection. On one hand, if VIP can protect the mitochondrial function during infection, it may enhance the protective barrier to infection, both via protection against apoptosis and via increased mucus secretion. On the other hand, as VIP has anti-inflammatory properties, this may impede the pathogen clearance as it has been demonstrated that for example IFNγ plays a role during clearance of infection [40]. To assess if VIP can restore the cytokine induced mitochondrial dysfunction, we utilized an *in vivo*-like *in vitro* mucosal cell line model that forms a polarized epithelial surface and secretes a mucus layer [41]. *C*. *rodentium* and *E*.*coli* infections have similar effects on mitochondrial function in this model [14]. In the murine *C*. *rodentium* infection model, we assessed the impact of VIP on colitis, mitochondrial function, pathogen- and colitis burden. Since our results demonstrated a decrease in colonic VIP after infection, we hypothesized that restoring VIP levels may yield protection. We started the VIP administration post infection, due to that this is the most likely situation for a therapeutic regimen under non-experimental conditions.

## Materials and methods

### Animals

Specific-pathogen-free C57BL/6 mice (6–8 weeks old, male) purchased from Taconic (England) or Charles River (Germany) were housed under pathogen-free conditions in individually ventilated cages at the Laboratory for Experimental Biomedicine (EBM) at Sahlgrenska Academy, Gothenburg. The animals were monitored daily and had free access to food and water.

### Ethics statement

All procedures were reviewed and approved by the Göteborgs Djurförsöksetiska Nämnd (Ethic No. 261/09, Djurskyddsförordningen DFS 2004:4).

### Culture of the *in vitro* colonic mucosal model

**Fig 1:**
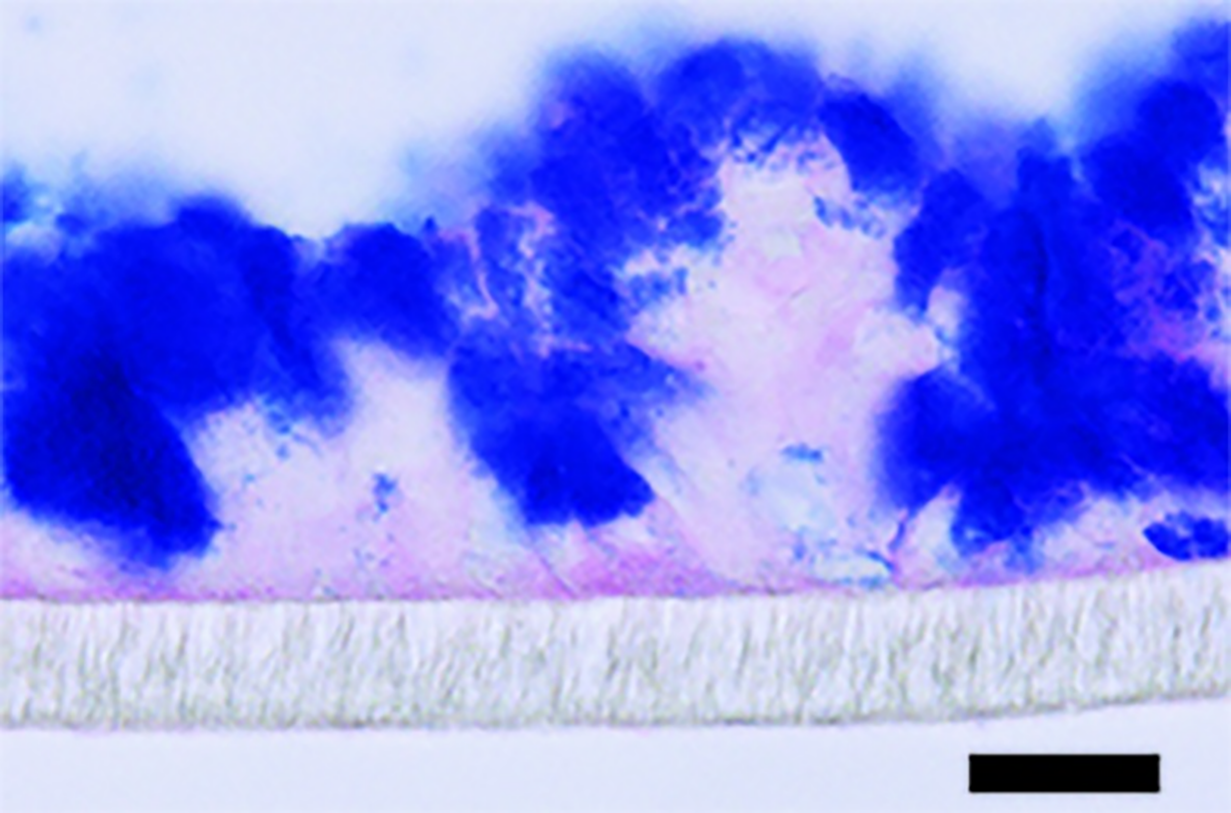
*In vitro* mucosal model. HT29 MTX-E12 cells cultured for 28 days post confluency under semi-wet interface with mechanical stimulation treated with DAPT for the first six days. The membranes were fixed in Methanolic Carnoy’s and stained with PAS/Alcian blue. The image was taken at 400x and the scale bar represent 20 μm.

The *in vitro* mucosal surface (Fig 1) was based on the colorectal adenocarcinoma HT29 MTX-E12 cell line (originated from HT29, American Type Culture Collection, USA, and was selected by growth adaptation to methotrexate and goblet cell morphology) and produced as previously described [14]. For expansion, the human intestinal cell line HT29 MTX-E12 was cultured (at 37°C, 5% CO2−95% air) in RPMI containing 10% (v/v) FCS, 1% 5000 U (v/v) penicillin-streptomycin (Lonza). To form the *in vivo* like *in vitro* mucosal surface [41], 7.5 x 10^4^ cells in 200 *μ*l of RPMI containing penicillin-streptomycin and 10% (v/v) FCS were added to the apical side of Snapwell membranes (0.4 mm pores) with 12 mm diameter (Corning). Four to 6 days later (when cells became confluent), the cells were subjected to semi-wet interface culture with continuous rocking, 50 μl of media in the apical compartment and 2 ml in the basolateral compartment for 28 days. Basolateral media was changed daily and supplemented with 10 mM *N*-[(3,5-Difluorophenyl)acetyl]-L-alanyl-2-phenyl]glycine-1,1-dimethylethyl ester (DAPT, Sigma-Aldrich) for the first 6 days and thereafter refreshed every two days.

### Bacterial culture

*C*. *rodentium* strain ICC169 was grown for 20 h at 37˚C on MacConkey agar (Oxoid).

### Infection and treatments

#### Infection and cytokine treatments *in vitro*

Starting on day 28 post confluency, the above described *in vitro* mucosal surfaces were subjected to VIP and cytokine treatment for 96 h. The 96 h treatment duration was chosen to mimic *in vivo* exposure during *C*. *rodentium* infection [14]. The cultures were exposed to IFN*γ* (10ng/ml) and TNF*α* (10ng/ml) in the presence and absence of VIP (0.5nM and 10 nM, Sigma-Aldrich). The concentrations of the cytokines, IFN*γ* (10ng/ml) and TNF*α* (10ng/ml) and VIP (0.5nM and 10nM) were chosen based on previous work [42–47]. Every 24 h, the antibiotic-free RPMI containing 10% FBS and VIP and/or cytokines was changed. To infect the *in vitro* mucosal surface, 10 μl of *C*. *rodentium* suspension (OD _410_ = 2, CFU, multiplicity of infection of 10:1) in sterile PBS was added to the apical side of the membrane 24 h before terminating the experiment.

#### Infection and VIP treatments *in vivo*

Male C57BL/6 mice were infected with 100 μl of the bacterial suspension [5 x 10^9^ colony forming units (CFU) in Luria-Bertani broth] by oral gavage. At day 10, 14 and 19 post infection, control and infected mice were anesthetized with isoflurane and killed by cervical dislocation. The most distal 2 cm of colon were collected: the most proximal cm was collected for isolation of mitochondria in ice cold imidazole buffer (50 mM, pH 7.4) and the most distal cm was put in fresh Carnoy’s methanol fixative (60% dry methanol, 30% chloroform, 10% glacial acetic acid).

VIP treatment: For all *in vivo* experiments with VIP treatment, an intraperitoneal (i.p.) saline (0.9%) administered dose of 0.5 nmol VIP per mouse/day was chosen as this dose had previously been shown to be the best dose for alleviating *C*. *rodentium* induced colitis, and this dose had no effect on body weight or behavior of uninfected mice [34]. For the first batch of experiments, one group received VIP daily from day 5 to 10 and was sacrificed on day 10 post-infection (n = 4) while the other group was administered VIP from day 5 to 14 and sacrificed on day 14 post-infection (n = 4). For the next batch of experiments, all mice were harvested on day 14 post-infection: one group was injected with VIP daily from day 5 to day 14 post-infection (n = 3), the second group from day 5 to day 10 (n = 4) and the third group from day 10 to day 14 (n = 4). Control mice received saline alone. Three mice died among the mice that were harvested on day 14 post infection: one in the group that was administered VIP day 5–10 and two in the group that were administered VIP day 10–14, rendering these groups small. For ethical reasons these treatment regimens were not repeated, and the group that only contained two surviving mice were therefore excluded from statistical analysis.

### Histology

For colitis analysis, 5 μm sections of Carnoy’s fixed tissue were stained with haematoxylin/eosin and the entire section was systematically scored for: crypt abscesses (0–3), aberrant crypt architecture (0–3), tissue damage (0–3), increased crypt length (0–3), goblet cell depletion (0–3), lamina propria neutrophil counts (0–3) and inflammatory cell infiltration (0–3) in a blinded fashion. The colitis score represents the sum of the individual scores for crypt abscesses, aberrant crypt architecture, tissue damage, increased crypt length, lamina propria neutrophil counts and inflammatory cell infiltration.

The goblet cell depletion scores were confirmed using PAS/Alcian blue stain, as previously described [14]: after immersion in 100% ethanol for 10 min, sections were rinsed in water for 10 min, immersed in 3% acetic acid for 2 min and stained in 1% Alcian Blue 8GX in 3% acetic acid (pH 2.5) for 2.5 h. Sections were then immersed in 3% acetic acid and rinsed in water for 10 min. The sections were then oxidized in 1% periodic acid (in water) for 10 min, washed in water for 5 min, immersed in Schiff's reagent for 10 min, rinsed again in water for 5 min and then three times in 0.5% sodium meta-bisulfite before a final water wash.

### Immunohistochemistry

Immunohistochemistry was performed as previously described [14]. Antigen retrieval using Dako target retrieval solution, pH 9.0 (S2367, Dako) was performed for 30 min (murine samples) or 15 min (*in vitro* mucosal surface) at 95˚C, slides were then cooled for 40 min in room temperature. 3% hydrogen peroxide in PBS was used to block endogenous peroxidase activity (15 min) and non-specific antibody binding blocked by incubation in Dako protein block serum-free reagent (X0909, Dako) for 30 min, room temperature, prior to incubation with primary antibodies. The following rabbit polyclonal primary antibodies for subunits of the mitochondrial respiratory enzyme complexes were used: complex I (anti-NADH dehydrogenase subunit 6, MTND6, orb6548, Biorbyt), complex-II (anti-Succinate dehydrogenase subunit A, SDHA, ab86932, Abcam), complex-III (anti-Ubiquinol-cytochrome c reductase complex cytochrome c1 subunit, CYC1, NBP1-86872, Novus Biologicals), complex-IV (anti-Cytochrome c oxidase subunit VIc, CCO-VIc, ab150422, Abcam), Caspase-3 (activated form, ab4051, Abcam), 3-Nitrotyrosine (AB5411, Millipore) and VIP (ab8556, Abcam) at optimal dilutions of 1:2000, 1:200, 1:2000, 1:2000, 1:500, 1:500 and 1:200 respectively, in antibody diluent (Dako, S0809). Incubation with the complex-II (SDHA) and Caspase-3 primary antibodies was for 16 h at 4˚C; incubation with VIP complex I, III, IV and 3-Nitrotyrosine antibodies for 3 h at 23˚C. Sections were then washed four times in PBS containing 0.05% Tween 20 and incubated with horseradish peroxidase (HRP) labelled anti-rabbit polymer (Dianova GmbH, PT03-L) for 15 min. Bound polymer was visualized using 0.05% 3,3’-diaminobenzidine hydrochloride (DAB) chromogen (CD-12, Dako) for 10 min, and tissue was counterstained with haematoxylin. Scoring of staining intensity (scale of 0–5) was performed blinded, and scores of key samples were verified by a second blinded independent observer. Results plotted in the graphs represent the average score of 1 cm distal colon.

### Ussing chamber experiments on mouse colon

Experiments were performed as previously described [48]. Briefly, dissected tissue specimens were flushed with cold KREB’s buffer to remove fecal material, kept on ice in KREB’s buffer for 30 min, opened along the mesenteric border and mounted in the Ussing chambers (exposed area 0.25 cm^2^). Both the serosal and mucosal chambers were filled with 2 ml KREB’s buffer and gassed with 95% O_2_ and 5% CO_2_ at 37 ºC, pH 7.4. The serosal solution also contained 5.1 mM Na-L-Glutamate, 5.7 mM Na-Pyruvate and 10 mM D-Glucose (Riedel-Haen AG) and the mucosal solution contained 5.13 mM Na-L-Glutamate (Merck), 5.7 mM Na-Pyruvate (Sigma-Aldrich) and 10 mM D-Mannitol (BDH Laboratory supplies).

### Bacterial localization and Muc2 expression in tissue

#### Bacterial localization

The slides were deparaffinized and dehydrated in 95% ethanol. Hybridization solution (40% (v/v) formamide, 0.1% SDS, 0.9 M NaCl, 20 mM Tris-HCl pH 7.4) containing 10 ng/μl of Cy3.5 5´ labeled eubacteria-specific probe (5´GCTGCCTCCCGTAGGAGT-3´) was added to tissue sections and incubated at 45°C in a humidified chamber containing 40% formamide over-night (~18h). Subsequently, the slides were washed with 0.9 M NaCl, 20 mM Tris-HCl pH 7.4 at 50°C for 20 minutes, followed by a brief submersion in room temperature PBS. For co-staining, sections were incubated with monoclonal rabbit-anti-*E*. *coli* O152 antiserum (Denka Seiken) diluted 1:100 at 4°C over-night followed by another over-night incubation with Alexa Fluor 488-conjugated goat anti-rabbit diluted 1:100 at 4°C, rinses and reagents were used as mentioned above for immunofluorescence. DAPI was used to visualize DNA as described above.

#### Muc2 staining

Paraffin embedded sections were deparaffinized and rehydrated. Sections were heated in 0.01 M citric acid buffer pH 6 for 20 min at 99°C. Non-specific binding was blocked by 5% FBS and the sections were incubated O/N at 4°C with an antibody recognizing the MUC2 mucin (polyclonal rabbit-anti-MUC2C3, kind gift of Professor G. Hansson, University of Gothenburg) diluted (1:1000) in blocking buffer. Slides were then incubated for 1 h at RT with Alexa Fluor 488-conjugated anti-rabbit immunoglobulin (1:500, Life Technologies). DAPI was used to visualize DNA as described above. Pictures were captured with an Eclipse 90i fluorescence microscope (Nikon).

### Isolation of the mitochondrial fraction

Murine colon: Mitochondria were isolated as previously described [14]. Briefly, epithelial scrapings from specimens collected in cold imidazole buffer (50 mM, pH 7.4) were suspended in 5 ml of homogenizing buffer A [225 mM mannitol, 75 mM sucrose, 5 mM 4-(2-hydroxyethyl)-1-piperazineethanesulfonic acid (HEPES), 1 mM ethylene glycol tetraacetic acid (EGTA), 1 mg/ml bovine serum albumin (BSA, pH 7.4)] and homogenised. The homogenate was brought to 15 ml with the same buffer and centrifuged for 10 min at 1000 *g* and 4˚C. The supernatant was saved, the pellet resuspended in homogenizing buffer A and centrifuged for 10 min at 1000 *g* and 4˚C. Supernatants from these steps were pooled and centrifuged for 10 min at 1000 *g* and 4˚C. The pellet was used for mitochondrial isolation and the supernatant for the nitrite assay. The pellet was resuspended in homogenization buffer A without BSA and EGTA and centrifuged for 10 min at 1000 *g* and 4˚C. For measurement of mitochondrial respiratory complex activities, mitochondria were resuspended in 50 mM phosphate buffer, pH 7.4; for measurement of membrane potential, mitochondrial ATP and phosphorylation capacity, the mitochondrial pellet was resuspended in isotonic buffer (145 mM KCl, 50 mM sucrose, 1 mM EGTA, 1 mM magnesium chloride, 10 mM phosphate buffer, pH 7.4). All aliquots were kept frozen at -20°C and used within a week.

*In vitro* mucosal surface: Mitochondria were isolated as previously described [14]. Briefly, cells from 12 membranes, pooled into 3 replicates (4 membranes per sample) per combination of treatment regimen were harvested in culture medium, pelleted, and washed with homogenization buffer B [250 mM sucrose, 1mM Tris-HCI, l mM ethylene diaminetetraacetic acid (EDTA, Sigma-Aldrich), 1 mg/ml of BSA, pH 7.4] at 4˚C. The cells were then resuspended in 5 ml of cold homogenization buffer B, homogenised to disrupt at least 95% of the cells and centrifuged for 10 min at 1000 *g* and 4˚C. The supernatant was saved, the pellet resuspended and centrifuged again for 10 min at 1000 *g* and 4˚C. The supernatants from these steps were pooled and centrifuged for 10 min at 1000 *g* and 4˚C. The supernatant obtained was stored at -20˚C for the nitrite assay and the pellet resuspended in homogenization B buffer containing digitonin (0.02%), centrifuged at 10000 *g* and resuspended in a buffer appropriate for further experimentation (same as for colon tissue, above).

### Measuring mitochondrial respiratory enzyme complex activities

NADH-ferricyanide reductase (complex-I) activity was measured in accordance with the method of Hatefi [49] with ferricyanide as the electron acceptor in a system containing 0.6 mM ferricyanide, 0.17 mM NADH, and 0.1% v/v Triton X-100 in 50 mM phosphate buffer, pH 7.4 at 30˚C. Addition of mitochondrial suspension (10 μg protein) initiated the reaction and the rate of oxidation of NADH was measured by the decrease in absorbance at 340 nm.

The activity of succinate cytochrome c reductase (complex II–III) was measured by analyzing the succinate supported reduction of ferricytochrome c to ferrocytochrome c at 550 nm in an assay mixture containing 100 mM phosphate buffer, 1 mM KCN, 2 mM succinate, 0.3 mM EDTA and 1.2 mg/ml cytochrome c (Sigma-Aldrich) in 0.4 ml [50]. Adding mitochondrial suspension (10 μg protein) to the sample cuvette initiated the reaction and the assay was repeated with (10 μM) antimycin (Sigma-Aldrich) to determine the inhibitor sensitive rate. The results were expressed as nmoles of cytochrome c reduced/min/mg protein.

The activity of cytochrome c oxidase (complex IV) was measured by quantifying the rate of decrease of absorbance at 550 nm at room temperature following the oxidation of reduced cytochrome c (50 *μ*M) in 10 mM phosphate buffer, pH 7.4 [51]. Ferricyanide (1 mM) was added to oxidize ferrocytochrome c in the blank cuvette and addition of mitochondrial suspension (10 μg) in the sample cuvette initiated the reaction. The results were expressed as nmoles of cytochrome c oxidised/min/mg protein.

### Assessing mitochondrial phosphorylation capacity

Phosphate utilization was measured as previously described [52]. Briefly, 25 μl mitochondrial suspension was diluted into a solution containing 10 mM HEPES, 125 mM KCl, 75 mM sucrose, 1 mM MgCl_2_, 2 mM phosphate, 0.1 mM EGTA, 0.3% BSA, 0.5 mM ADP (Sigma-Aldrich), 5 mM pyruvate, 10 mM succinate and 10 mM glucose, 5 units of hexokinase (Sigma-Aldrich) added, in total volume of 250 μl, and incubated for 30 min at 37˚C. The reaction was terminated by 5% ice cold trichloroacetic acid (TCA) and the inorganic phosphate was determined spectrophotometrically. Inorganic phosphate content was also assayed in a 0 min sample, where hexokinase addition was immediately followed by treatment with 5% cold TCA. Hexokinase and glucose in the reaction mixture acted as a trap for ATP to maintain the level of ADP in the system and to prevent release of free inorganic phosphate from ATP by phosphatases.

### Assessing ATP synthesis

ATP content was determined in aliquots of mitochondrial suspension using a commercial ATP assay kit (ab83355, Abcam).

### Assessing mitochondrial transmembrane potential

Murine colon: mitochondrial suspensions were incubated for 30 min at 37˚C in isotonic buffer A containing 10 mM succinate, 10 mM pyruvate, 1 mM ADP and 5 μM JC-1 (5,5',6,6'-tetrachloro-1,1',3,3'- tetraethyl benzimidazolylcarbocyanine iodide, CS0760, Sigma-Aldrich). The dyed mitochondria were then collected by centrifugation, washed with buffer A and resuspended in the same buffer and fluorescence intensity read (λex 490 nm, λem 590 nm).

*In vitro* mucosal surface: Mitochondrial transmembrane potential in intact cells was measured using a kit (ab113852, Abcam) in accordance with the manufacturer’s instructions.

### Protein concentration assessment

The method of Lowry [53] was used to determine protein concentration after solubilizing samples in 1% SDS.

### Statistical analysis

All analyses were performed using GraphPad Prism (version 3·0). Differences between groups were assessed using one-way analysis of variance (ANOVA) with Student Newman-Keuls Multiple Comparison test. Values were expressed as mean ± S.E.M and differences were considered significant if p < 0.05.

## Results

### Mucosal VIP decrease during murine *C*. *rodentium* infection with a similar time dependency as measurements reflecting mitochondrial function and epithelial integrity

**Fig 2:**
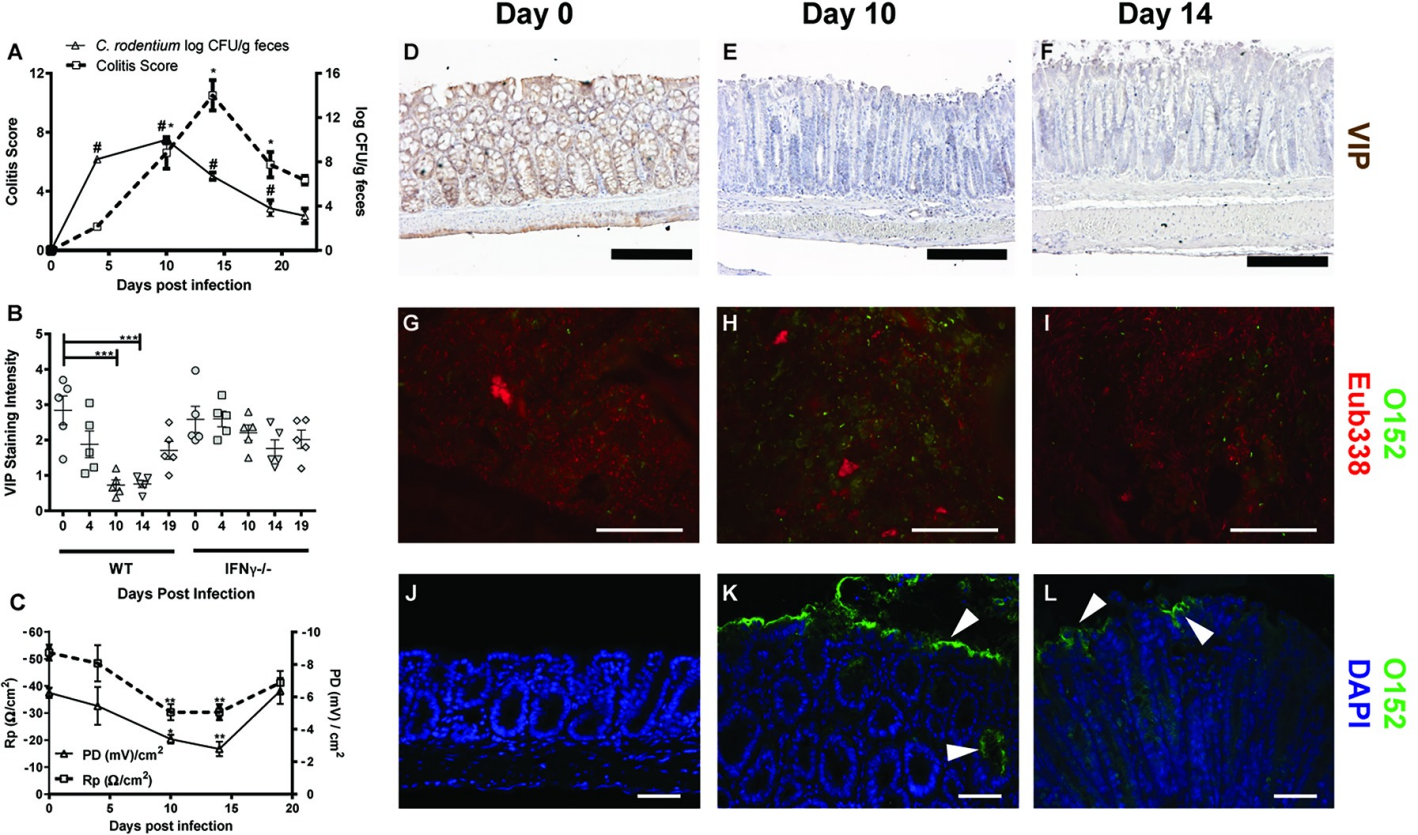
*C*. *rodentium* density, colonic epithelial health status and VIP staining intensity in the murine distal colon during infection. A: Fecal *C*. *rodentium* density and total colitis score: *C*. *rodentium* CFU in fecal pellets from individual mice. The colitis score is presented as the sum of the scores from tissue damage, crypt architecture, crypt length, crypt abscesses, goblet cell depletion, inflammatory cell infiltration and neutrophils in lamina propria. (B) Semi-quantification (visually scored 0–5) of the VIP staining intensity in the murine distal colon of wild type (WT) and IFN-γ^-/-^ mice. (C) Colonic epithelial health status measuring Rp (Ω/cm^2^) and PD (mV/cm^2^) of mice following day 4, 10, 14 and 19 post-infection in wild type mice infected with *C*. *rodentium*. (D-F) Representative images of distal colonic specimens immunohistochemically stained for VIP (brown). Scale bar 200 μm, magnification x200. (G-L) Colonic tissue sections co-stained with a general eubacterial probe (Eub338, red) and an antibody against *E*. *coli* lipopolysaccharide antigen O152 (green), which also stains *C*. *rodentium*. (G-I) Representative images of feces (scale bar 50 μm, magnification x400) and (J-L) epithelial surface (scale bar 50 μm, magnification x200). Statistics: data are presented as mean ± S.E.M. and analyzed using ANOVA with Student Newman-Keuls Multiple Comparison post hoc test: * *P*<0.05, ** *P*<0.01*** *P* <0.001 vs. control. (n = 5 mice/group). The experiments were performed twice.

In line with previous studies [54], the highest *C*. *rodentium* density in the feces of infected C57BL/6 wild type (WT) mice occurred around day 10, tended to decrease at day 14 and by day 19 and 22 post infection the number of *C*. *rodentium* in feces had decreased by log 5–6 to 100–1000 CFU/g feces (Fig 2A). The amount of *E*. *coli* lipopolysaccharide O152 antigen positive bacteria (also found in *C*. *rodentium*) compared to total bacteria in feces mirrored the fecal counts, with few O152 positive bacteria present before infection, large amounts present by day 10 and decreasing levels by day 14, and the proportion of 0152 negative eubacteria followed the opposite pattern (Fig 2G–1). There were no O152 positive bacteria detected in contact with the epithelium in uninfected animals, suggesting that the large amounts present at the epithelial surface by day 10 and 14 were indeed *C*. *rodentium* (Fig 2J–2L), whereas virtually no 0152 negative eubacteria were detected in contact with the epithelium at any time point. In non-infected WT mice, VIP was observed in the mucosa, submucosa and in the muscular layer comprised of circular and longitudinal muscles (Fig 2D). This distribution of VIP immunoreactivity is in line with previously published reports [34, 55–56], and also similar to the previously published localization of the mitochondrial complexes [14]. *C*. *rodentium* infection caused a gradual decline in VIP staining with maximum loss of VIP on day 10 and 14 post-infection, featuring marked reduction in immunoreactivity especially in the mucosal and submucosal region (p<0.001, Fig 2B, 2E and 2F). Furthermore, VIP levels were not significantly affected in IFNγ deficient infected mice (Fig 2B), paralleling that mitochondrial function was less affected in these mice than in WT [14]. This suggests that VIP levels, similarly to mitochondrial function, may be more affected by the cytokine environment than pathogen density, as we previously have shown that the *C*. *rodentium* density is similar between WT and IFNγ deficient infected mice at day 10 post infection [14]. The pattern of decreased VIP stain had a time dependency similar to the changes that occur in mitochondrial phosphorylation capacity [14], transepithelial resistance (Rp) and potential difference (PD, Fig 2C) [47]. To uphold PD requires sufficient ATP levels, ion channel activities and active transport of ions across the epithelium, whereas Rp reflects the electrical resistance of tight junctions. Since these factors co-varied, we decided to investigate if VIP could counteract the previously described negative effects of infection and associated pro-inflammatory cytokines on mitochondria [14].

### *In vitro* reduction of the enzymatic activity of the mitochondrial respiratory complexes I and IV caused by IFNγ, TNFα and *C*. *rodentium* infection was alleviated by VIP

We previously demonstrated that out of the many cytokines induced during *C*. *rodentium* infection, IFNγ and TNFα had the most severe effects on mitochondrial function, especially when combined [14]. Here we utilized an *in vivo*-like *in vitro* human colonic mucosal surface with a three-dimensional architecture, functional tight junctions, polarized cells and mucus secretion [41], to investigate the relation between VIP, these cytokines, infection and mitochondrial function. *C*. *rodentium* infection, IFNγ and TNFα did not have detectable effects on the endogenous VIP levels in this model, possibly due to that the levels were so low to start with (immunohistochemical staining score of 1 or below). Similarly, the mitochondrial parameters measured below were similar in *in vitro* mucosal surfaces with and without VIP treatment in the absence of other stimuli (Table 1).

**Table 1:** Mitochondrial functional parameters observed in control and VIP treated groups (0.5 and 10 nM) in *in vitro* mucosal cell line model. Values presented are mean ± S.E.M (n = 4).

Parameters	Control	VIP (0.5 nM)	VIP (10 nM)
Complex I Activity (nmoles of NADH oxidized/min/mg protein)	1190 ± 88.79	1220 ± 73.48	1150 ± 127.7
Complex II-III Activity (nmoles of cytochrome c reduced/min/mg protein)	110 ± 4.54	104.3 ± 3.74	102 ± 2.55
Complex IV Activity (nmoles of reduced cytochrome c oxidized/min/mg protein)	447.5 ± 15.61	438.5 ± 19.09	439.5 ± 6.021
Mitochondrial Phosphorylation (nmoles of Pi utilised/mg protein/min)	98.50 ± 9.023	100.8 ± 6.96	97.50 ± 4.62
Mitochondrial Membrane Potential (Fluorescence Intensity / 100mg protein)	710.33 ± 14.72	698.66 ± 6.86	701.34 ± 9.28
Mitochondrial ATP Generation (nmole of ATP/mg of protein)	4.26 ± 0.216	4.10 ± 0.170	4.06 ± 0.232

**Fig 3:**
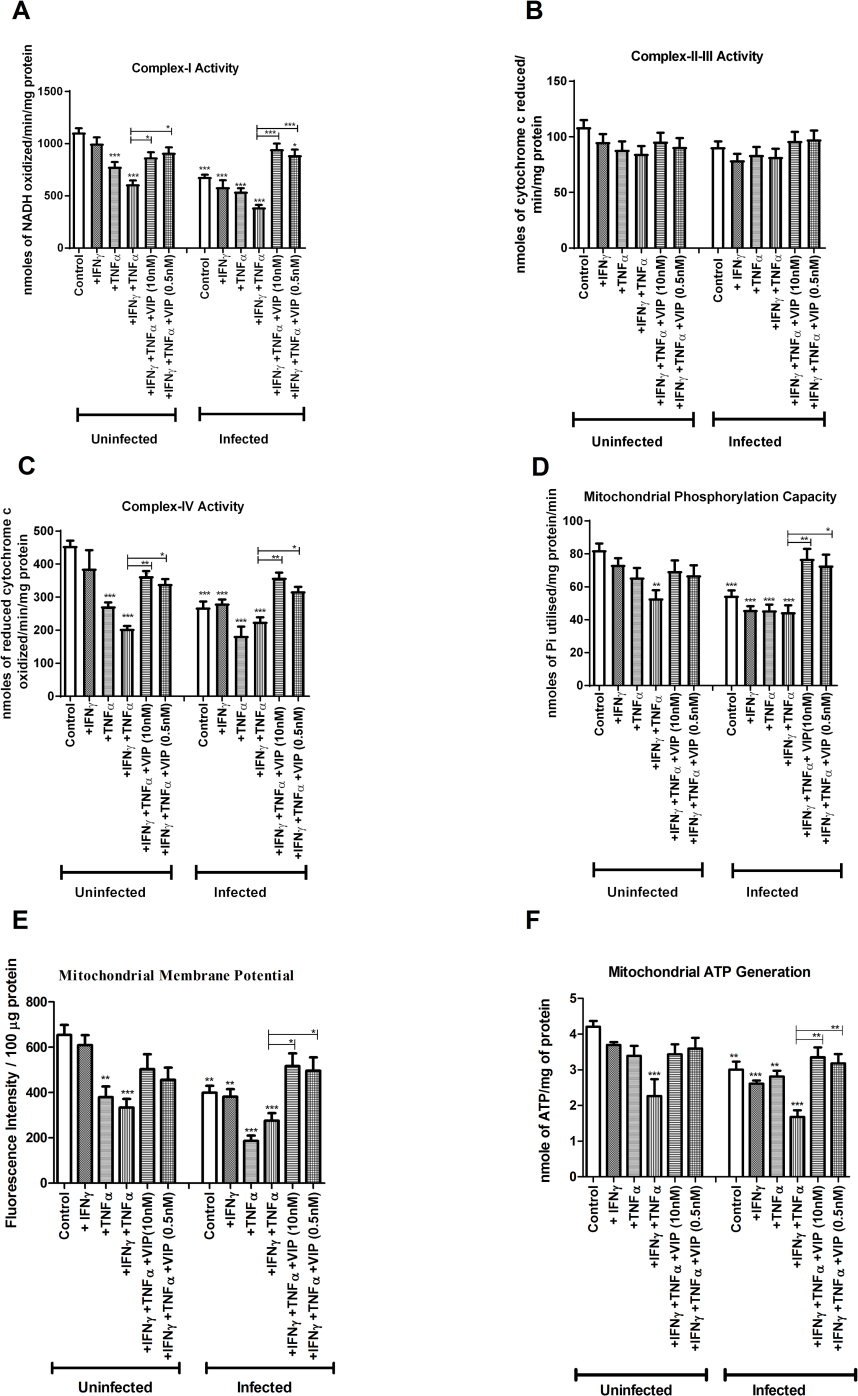
In vitro effects of VIP on mitochondrial dysfunction caused by *C*. *rodentium* infection and cytokines. An in vivo like *in vitro* intestinal mucosal model was treated with cytokines and infected in the presence and absence of VIP, and then six aspects of mitochondrial function were analyzed: (A) complex-I activity (B) complex-II-III activity (C) complex-IV activity (D) mitochondrial phosphorylation capacity (E) mitochondrial membrane potential and (F) mitochondrial ATP generation. Statistics: data are presented as mean ± S.E.M. (n = 4, biological replicates, results were pooled from two separate experiments) and analyzed by ANOVA with Student Newman-Keuls Multiple Comparison post hoc test: * *P*<0.05, ** *P*<0.01, *** *P*<0.001.

VIP at both doses (10 nM and 0.5 nM) reversed the combined inhibitory impact of IFNγ and TNFα on complex-I activity both in uninfected (10 nM: -45% to -18%, p<0.05; 0.5 nM: -45% to -17%, p<0.05, Fig 3A) and infected conditions (10 nM: -65% to -14%, p<0.001; 0.5 nM: -65% to -20%, p<0.001, Fig 3A). Similarly both doses of VIP also protected against the combined impact of IFNγ and TNFα on complex-IV activity in both uninfected (10 nM: -50% to -20%, p<0.01; 0.5 nM: -50% to -25%, p<0.05, Fig 3C) and infected conditions (10 nM: -55% to -21%, p<0.01; 0.5 nM: -55% to -30%, p<0.05, Fig 3C). Complex II-III activity was not affected by infection, cytokines or VIP (Fig 3B).

**Fig 4:**
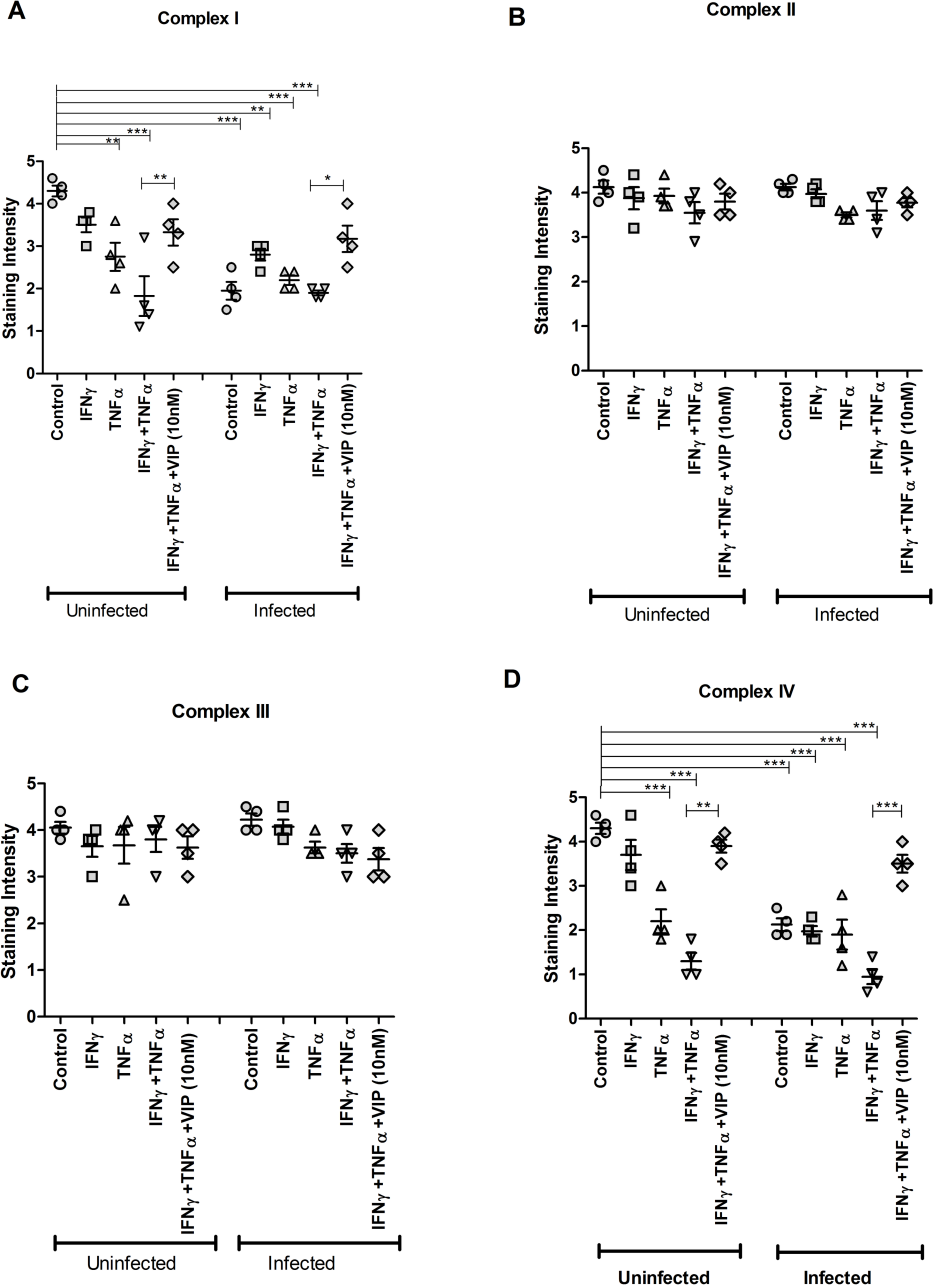
Effects of VIP on restoring the decreased levels of mitochondrial respiratory enzyme complexes caused by cytokines and *C*. *rodentium* infection *in vitro*. An in vivo like *in vitro* intestinal mucosal model was treated with cytokines and infected in the presence and absence of VIP, stained immunohistochemically and the intensity of the stain was scored. (A) Semi-quantification for complex I (MTND6 antibody), (B) complex-II (SDHA antibody) (C) complex-III (CYC1 antibody) and (D) complex-IV (CCO-VIc antibody). Statistics: data are presented as mean ± S.E.M. (n = 4, biological replicates, results were pooled from two separate experiments) and analyzed by ANOVA with Student Newman-Keuls Multiple Comparison post hoc test: * *P*<0.05, ** *P*<0.01, *** *P*<0.001.

As previously described [14], infection with *C*. *rodentium* for 24 h resulted in loss of immunohistochemical staining in the *in vitro* mucosal surface for complex I and IV (p<0.001 vs p<0.001) but not for complex II and III (Fig 4). VIP treatment reversed the decreased *in situ* protein levels of complex-I (MTND6) and complex-IV (CCO-VIc) in response to TNFα and IFNγ under uninfected (p<0.01, Fig 4A and 4D) and infected (p<0.05 vs p<0.001, Fig 4A and 4D) conditions. Thus, the protein levels of the enzymes in the tissue mirrored the results from the enzymatic assays, and the results are thus not likely affected by the mitochondrial isolation process.

### *In vitro*, VIP counteracted the decreases in mitochondrial phosphorylation capacity, transmembrane potential and ATP generation caused by IFNγ, TNFα and *C*. *rodentium* infection

The mitochondrial phosphorylation capacity was decreased by IFNγ and TNFα in combination, both when accompanied by infection (-48%, p<0.001) and under un-infected conditions (-36%, p<0.01, Fig 3D). The impairment of the mitochondrial phosphorylation caused by infection and cytokines was relieved by VIP to a level similar to that of non-treated mucosal membranes (Fig 3D, p<0.01). Similarly, VIP alleviated the detrimental combined effect of infection, IFNγ and TNFα on mitochondrial membrane potential (10 nM: -57% to -21%, p<0.05; 0.5nM: -57% to -24%, p<0.05, Fig 3E) and revived the mitochondrial ATP generation from -60% to -20% at 10 nM concentration and from -60% to -28% at 0.5 nM concentration (p<0.01, Fig 3F).

### Murine infection with *C*. *rodentium* decreased the activities of enzyme complexes I and IV, which were alleviated by VIP treatment regimens that included the day 5–10 post infection period

Next, we investigated if the decrease of enzyme activity of mitochondrial complexes on day 10 and 14 post infection in mice was alleviated by treatment with VIP (0.5 nmol/ mouse/ day). We selected a range of therapeutic treatment regimens that all started at day 5, or after day 5, post infection (i.e. when infection was fully established and the pathogen shed with feces, Fig 2A). For mice harvested at day 10 post-infection, mice were treated with VIP from day 5 to day 10 whereas for day 14 post-infection group, three regimens of VIP treatment were examined: day 5–14, day 5–10 and day 10–14, to investigate if we could locate a therapeutic window during the time where endogenous VIP was low and VIP supplement may be beneficial for the mucosal surface, without detrimentally affecting the actions of the immune response to clear the infection.

**Fig 5:**
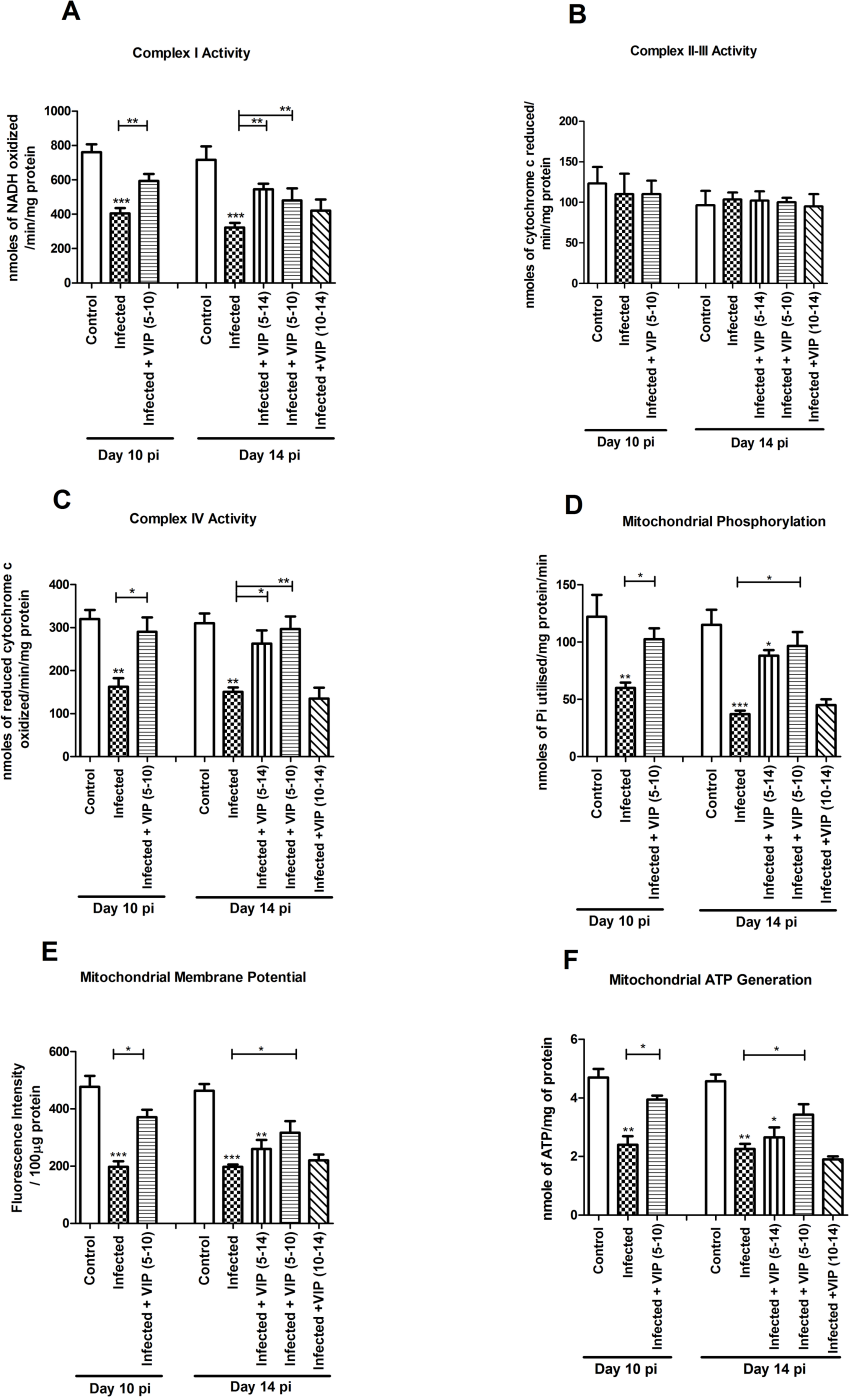
Mitochondrial function in the murine distal colon during *C*. *rodentium* infection with and without VIP administration. Mice were harvested at day 10 and 14 post infection. VIP was administered daily in three treatment groups of mice: mice treated with VIP for day 5–10 were harvested both at day 10 and 14, and mice treated from day 5 to 14 and from day 10 to 14 post-infection were harvested on day 14. (A) complex I activity (B) complex II-III activity (C) complex IV activity (D) mitochondrial phosphorylation capacity (E) mitochondrial membrane potential (F) mitochondrial ATP generation. Statistics: data are presented as mean ± S.E.M. and analyzed by ANOVA with Student Newman-Keuls Multiple Comparison post hoc test: * *P* <0.05, ** *P* <0.01, *** *P* <0.001 vs. control. (n = 2–7 mice/group). Of the mice harvested day 14 post infection, one mouse died in the group that was administered VIP day 5–10 and two in the group that were administered VIP day 10–14. Data about dead animals is not included in the graphs, and the group with only two mice remaining (VIP day 10–14) was omitted from the statistical analysis.

In mice infected for 10 days, complex-I activity decreased by 45% (p<0.01, Fig 5A). VIP treatment from day 5 to 10 restored the complex-I activity (-45% to -13%, p<0.01, Fig 5A) to an extent that it was not statistically different from the activity levels in non-infected controls. Complex-IV activity was reduced by 48% (p<0.01, Fig 5C). VIP treatment for day 5–10 alleviated the reduction in complex-IV activity (-48% to -5%, p<0.05, Fig 5C).

In mice infected for 14 days, complex-I activity was decreased by 60% (p<0.001, Fig 5A). VIP treatment for day 5–14 and day 5–10 alleviated the reduction in complex-I activity (-60% to -19%, p<0.01, and -60% to -24%, p<0.01, Fig 5A). Similarly, VIP treatment for day 5–10 and 5–14 alleviated the reduction in complex-IV activity (-53% to -7%, p<0.01, and -53% to -14%, p<0.05, Fig 5C). No decrease of enzymatic activity was observed for complex-II-III at any investigated time points, and VIP had no effect either (Fig 5B). In the day 10–14 VIP treatment cohort, two of the mice died, rendering the group too small to include in the statistical analysis, however, the mitochondrial parameters were very similar in this group as in the infected controls, suggesting that VIP treatment day 10–14 did not have any beneficial effects (Fig 5).

### *C*. *rodentium* infection caused reductions in phosphorylation capacity, mitochondrial transmembrane potential and ATP generation, which were alleviated by VIP treatments that included the day 5–10 post infection period

The mitochondrial phosphorylation capacity, measured by inorganic phosphate utilization, was reduced by 51% at day 10, and by 61% at day 14 (p<0.01, Fig 5D). VIP treatment for day 5–10 alleviated the reduction in mitochondrial phosphorylation capacity for both the day 10 (-51% to -8%, p<0.05, Fig 5D) and day 14 (-61% to -17%, p<0.05, Fig 5D) post infection groups. VIP treatment for day 5–14 also trended to alleviate the reduction in mitochondrial phosphorylation capacity (Fig 5D). Similarly, the mitochondrial transmembrane potential was reduced by 50% or more at both day 10 and 14 post infection (p<0.001, Fig 5E), and VIP treatment for day 5–10 alleviated the reduction at both time points (p<0.05, Fig 5E), whereas treatment for day 10–14 or 5–14 was less effective (Fig 5E). As a consequence, ATP generation was also hampered at day 10 and 14 post-infection (p<0.01, Fig 5F), and VIP treatment for day 5–10 alleviated the reduction at both time points (-46% to -8%, p<0.05, and -48% to -25%, p<0.05), whereas treatment for day 10–14 or 5–14 was less effective (Fig 5F).

### VIP treatment failed to alleviate colitis but tended to modify bacterial density and tissue localization

**Fig 6:**
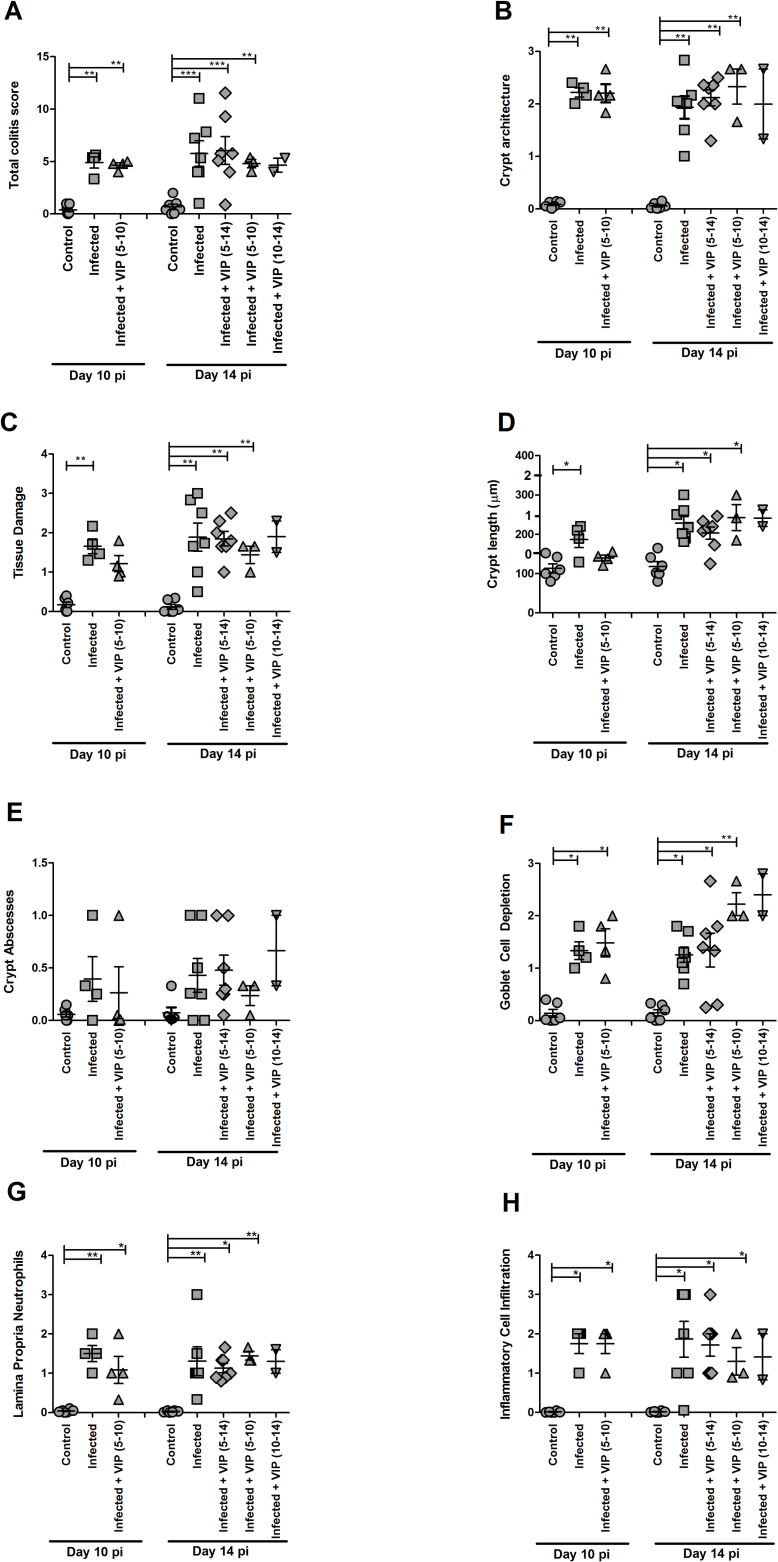
Effect of VIP on colitis during *C*. *rodentium* infection. A. The total colitis score is the sum of the scores present in A, B, C, D, E, G and H: The goblet cell depletion score (F) was not included, due to the fact that VIP is a mucus secretagogue. (B) crypt architecture, (C) tissue damage, (D) crypt length (the crypt lengths were translated into scores for incorporation into the total colitis score according to the numbers 1–3 on the y-axes), (E) crypt abscesses, (F) goblet cell depletion, (G) neutrophils in lamina propria and (H) infiltration of inflammatory cells. Statistics: data are presented as mean ± S.E.M. (n = 2–7 mice, as indicated by the individual symbols in the graph) and analyzed using ANOVA with Student Newman-Keuls Multiple Comparison post hoc test: * *P*<0.05, ** *P*<0.01, *** *P*<0.001 vs. control. Of the mice harvested day 14 post infection, one mouse died in the group that was administered VIP day 5–10 and two in the group that were administered VIP day 10–14. Data about dead animals is not included in the graphs, and the group with only two mice remaining (VIP day 10–14) was omitted from the statistical analysis.

**Fig 7:**
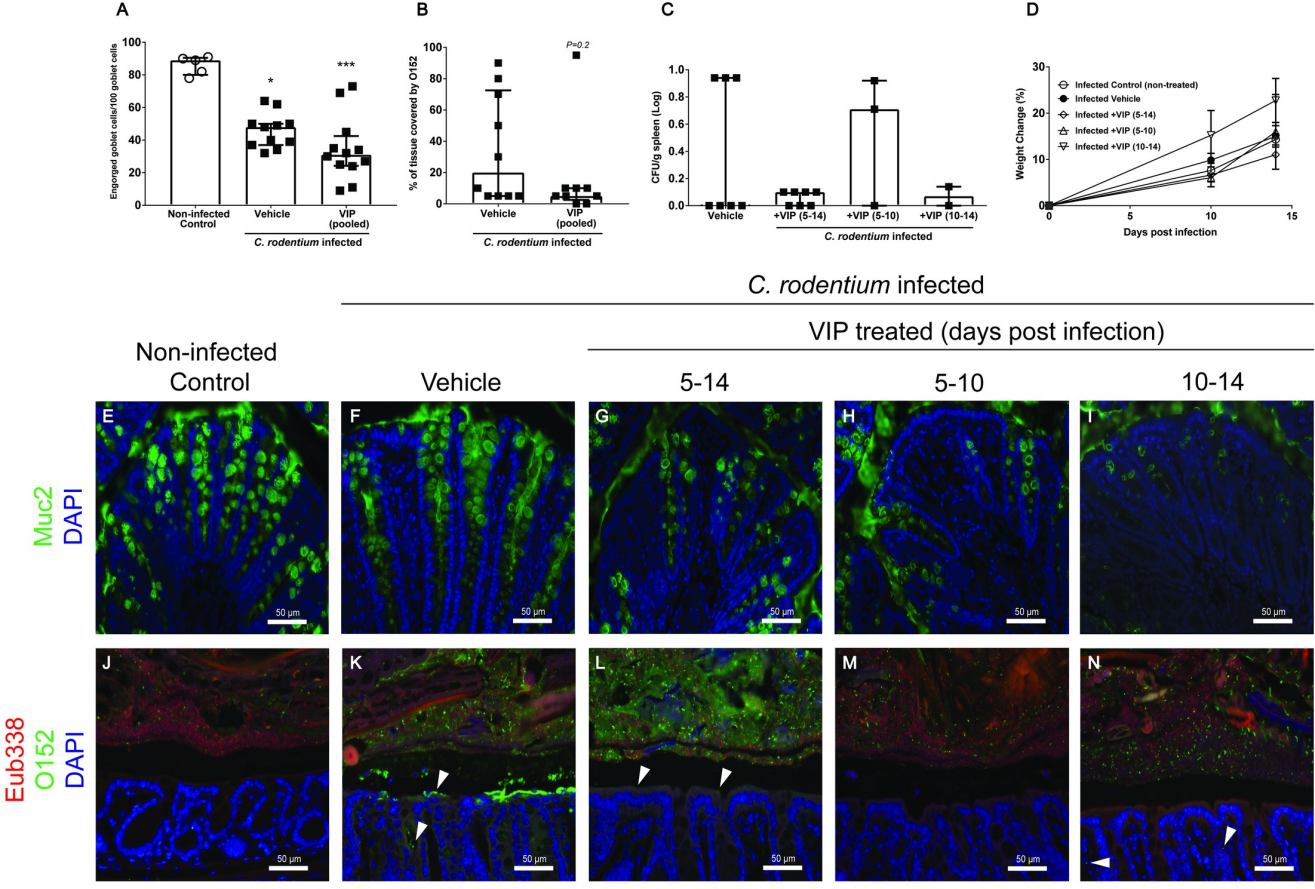
*C*. *rodentium* density and localization during VIP treatment at day 14 post-infection. Colonic tissue sections were stained with anti-Muc2 or co-stained with a general eubacterial probe and an antibody against *E*. *coli* lipopolysaccharide antigen O152, which also stains *C*. *rodentium*. (A) The number of engorged/Muc2 filled goblet cells per 100 goblet cells in the upper third of the epithelium, scores were pooled for the three VIP regimens. Statistics: Kruskal Wallis test. (B) Percentage of the epithelial surface covered by O152 positive bacteria, scores were pooled from the three VIP regimens. Statistics: Mann-Whitney U-test. (C) The amount of *C*. *rodentium* (CFU/g) in the spleen in VIP treated and non-treated infected mice. Each data point represents an individual mouse, and bars represent median and interquartile range (panels A-C). Of the mice harvested day 14 post infection, one mouse died in the group that was administered VIP day 5–10 and two in the group that were administered VIP day 10–14. (D) Change in body weights presented as mean±S.E.M. (E-N) Representative images (images were taken from sections that obtained the median score for each group) of colonic tissue sections stained for Muc2 (green) (E-I) or co-stained using the eubacterial probe (Eub338, red) and anti-O152 (green) (J-N) of non-infected control (E and J), infected vehicle treated (F and K), and infected VIP treated mice for day 5–14 (G and L), day 5–10 (H and M) and day 10–14 (I and N) (magnification x 200).

Infection with *C*. *rodentium* produced features typical of colitis (tissue damage, goblet cell depletion, increased crypt length, alteration of crypt architecture, neutrophils present in lamina propria) at day 10 and 14 post infection (p<0.05), but no VIP treatment regimen ameliorated the colitis (Fig 6). The level of Muc2, the main mucin making up the colonic mucus layer, decreased in infected vehicle- (p<0.05) and VIP treated mice compared to non-infected controls (p<0.001, Fig 7E–7I). Nevertheless, the Muc2 immunoreactivity of VIP treated mice did not differ from vehicle treated mice, and mirrored the goblet cell depletion scores (compare Fig 6F with 7A). VIP treatment tended to affect bacterial localization: VIP treated mice tended to have fewer *C*. *rodentium* in contact with the colonic epithelium (Fig 7B and 7J–7N, p = 0.2). Furthermore, there was a tendency towards that fewer VIP treated mice had *C*. *rodentium* in the spleen (2/12 VIP vs 3/7 control), although the difference was not statistically significant (Fig 7C). All mice gained weight during the infection (Fig 7D).

### *In vivo C*. *rodentium* infection increased apoptosis, which was counteracted by VIP regimens that included administration on day 5–10

**Fig 8:**
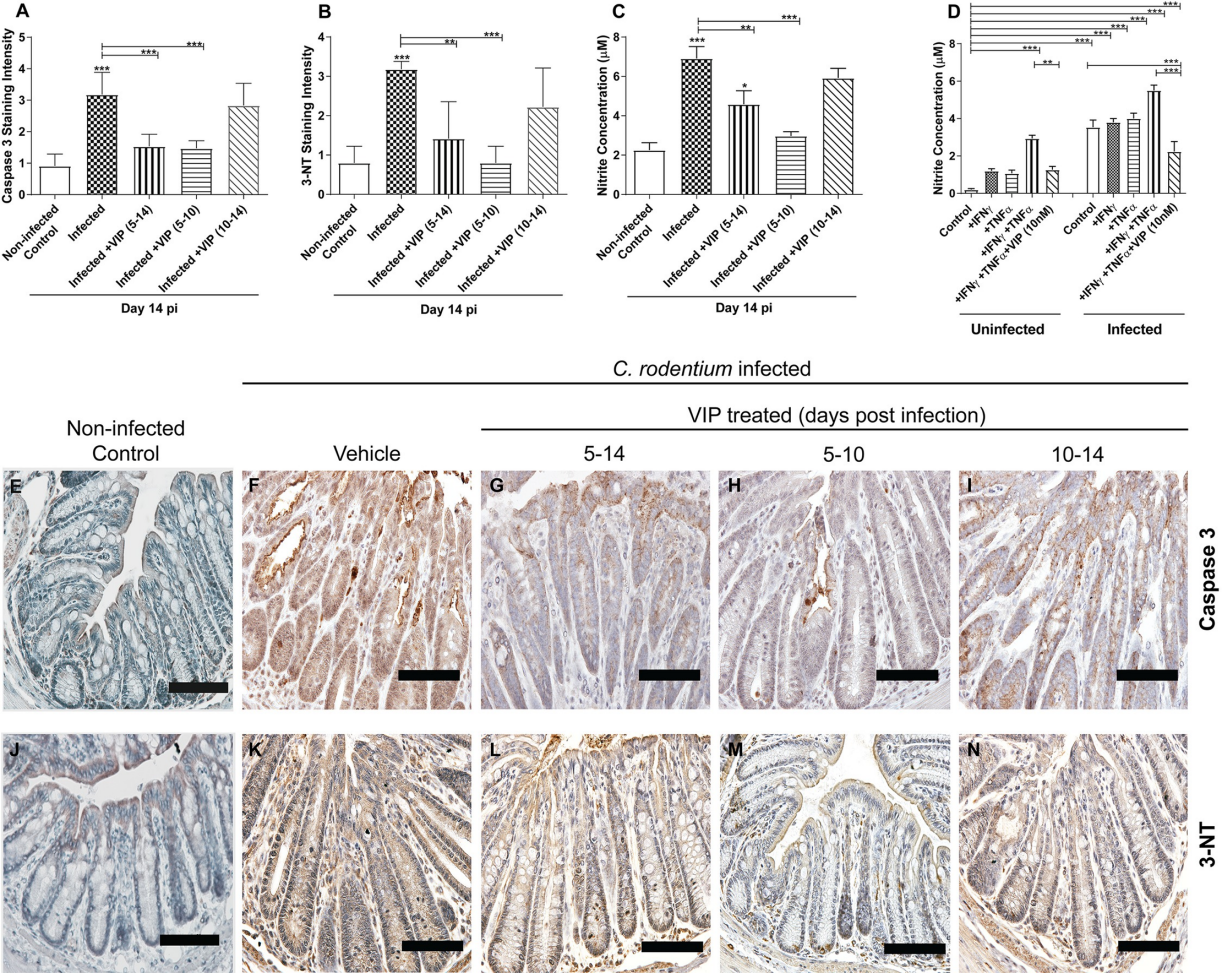
Caspase-3, 3-Nitrotyrosine (3-NT) staining scores and nitrite measurements at 14 day post-infection. (A) Semi-quantification of immunohistochemical Caspase-3 staining in distal colon from *C*. *rodentium* infected mice with and without VIP treatment (B) Semi-quantification of immunohistochemical 3-NT in distal colon from infected mice with and without VIP treatment. (C) Nitrite concentration in the murine distal colon of infected and VIP treated mice (n = 2–7 mice/group). Of the mice harvested day 14 post infection, one mouse died in the group that was administered VIP day 5–10 and two in the group that were administered VIP day 10–14. Data about dead animals is not included in the graphs, and the group with only two mice remaining (VIP day 10–14) was omitted from the statistical analysis. (D) Nitrite concentration in the *in vitro* mucosal intestinal model infected with *C*. *rodentium* and treated with cytokines in the presence and absence of VIP. (E-I) Representative photos showing caspase-3 tissue localization in distal colon from *C*. *rodentium* infected mice with and without VIP treatment. Note that although many cells in the tissue from infected mice stains pale brown, a study that performed both Caspase-3 and Terminal deoxynucleotidyl transferase dUTP Nick-End Labeling (TUNEL) assays in parallel to detect DNA degradation, demonstrated that the proportion of cells that are in late stage apoptosis correspond to the strongly stained cells, not the light brown cells [58]. (J-N) Representative photos showing 3-NT tissue localization in distal colon from *C*. *rodentium* infected mice with and without VIP treatment. Scale bar 200 μm, magnification x400. Statistics: data are presented as mean ± S.E.M. and analysed by ANOVA with Student Newman-Keuls Multiple Comparison post hoc test: * *P*<0.05, ** *P*<0.01, *** *P*<0.001.

*C*. *rodentium* infection leads to cell death and sloughing of cells [57], as supported by the fact that the level of the active cleaved form of caspase-3 (indicating apoptosis) increased in both crypts and luminal surface (Fig 8A). VIP treatment from day 5–14 and day 5–10 reverted the level of caspase-3 to baseline levels (p<0.01 and p<0.001) whereas VIP treatment from day 10–14 did not have any effect (Fig 8A). Pro-apoptotic molecules such as cytochrome c released from mitochondria activate the caspase-9-caspase-3 cascade, and the mitochondrial functional parameters indeed followed an opposite pattern to the caspase-3 stain. The induced apoptosis is likely due to oxidative damage, since the mitochondria targeting antioxidant MitoQ which scavenges peroxynitrite (ONOO^-^) that is generated when NO reacts with superoxide (O_2_^.-^), alleviates it [14].

### *C*. *rodentium* induced NO generation was alleviated by VIP both *in vivo* and *in vitro*

Immunostaining intensity for 3- Nitrotyrosine (3-NT, a marker for oxidative damage) increased during *in vivo* infection with *C*. *rodentium*. VIP treatment for day 5–14 and 5–10 alleviated the increase (p<0.01 and p<0.001), whereas VIP treatment for day 10–14 had less effect (Fig 8B), mirroring the effect on caspase-3 stain (Fig 8A). Another index of oxidative damage, NO_2_^-^ generation, also increased during both *in vivo* and *in vitro C*. *rodentium* infection (Fig 8C and 8D). *In vivo* VIP treatment for day 5–14 lessened NO_2_^-^ generation (3-fold to 2-fold, p<0.01, Fig 8C), and 5–10 reversed the NO_2_^-^ generation to an extent that it was not different from non-infected control animals (3-fold to 1.3-fold, p<0.001, Fig 8C), however, VIP treatment for day 10–14 appeared to have less effect (Fig 8C). The combined action of TNFα and IFNγ 14-folded the NO_2_^-^ generation in the non-infected *in vitro* mucosal membranes (p<0.001), and infection further increased it (p<0.001, Fig 8D). VIP (10 nM) alleviated the NO_2_^-^ generation induced by IFNγ and TNFα with and without infection (p<0.001, Fig 8D).

## Discussion

This study demonstrated that VIP decreased during murine *C*. *rodentium* infection in a cytokine dependent manner and that VIP supplementation alleviated the reduction of activity and levels of mitochondrial respiratory complexes I and IV, mitochondrial phosphorylation capacity, transmembrane potential and ATP generation caused by IFNγ, TNFα and *C*. *rodentium* infection in an *in vitro* mucosal surface. Similarly, VIP treatment regimens that included the day 5–10 post infection period alleviated decreases in enzyme complexes I and IV, phosphorylation capacity, mitochondrial transmembrane potential and ATP generation as well as increased apoptosis levels during murine infection with *C*. *rodentium*, demonstrate that the timing of the treatment is vital. However, although there was a tendency to decreased pathogen density in contact with the epithelium and in the spleen, VIP treatment failed to alleviate colitis. The protective actions of VIP on mitochondria and epithelial cells is likely due to decreased oxidative damage, as the *C*. *rodentium* infection induced NO was alleviated by VIP both *in vivo* and *in vitro*. Thus, therapeutic VIP administration to restore the decreased levels during infection had beneficial effects on epithelial cells and their mitochondria, but not on the overall infection outcome.

To investigate if VIP had a therapeutic value for infection, we selected to start treatment after day five, when infection is fully established. The same low dose of VIP (0.5 nmol/ mouse) was chosen for our murine study as VIP in higher doses (1 and 5 nmol/ mouse) failed to provide beneficial impact on *C*. *rodentium* infection induced colitis due to downregulation of receptors [34]. Although colitis or bacterial burden was not decreased, we did find beneficial effects on the epithelial cells and their mitochondria using regimens that included VIP administration day 5–10, whereas late (day 10–14) VIP treatments appeared less effective. In addition to being less effective, we also obtained indications that VIP administration during the latter period of infection had negative effects: two out of four mice receiving VIP during this period died, and although we have no hard data on this, the other mice in this group appeared less active than non-treated mice. The number of mice in the 5–10 and 10–14 day treatment cohorts were low (only three mice in one and two in the other, due to the fact that mice died in these groups) compared to the seven mice in total in the 5–14 day treatment. Thus, the results from the two short-term treatments are less certain. However, due to the adverse reaction and lack of benefits on the overall outcome of the infection in spite of clear benefits on the mitochondrial function, we did not consider it ethically responsible to perform further experiments to add numbers to these groups.

A previous study demonstrated that prophylactic VIP treatment decreased the *C*. *rodentium* induced colitis at 10 days post infection, although bacterial attachment was not affected [34]. Together with our data where treatment started day 10 post infection to mimic therapeutic treatment, this suggests that the timing of the VIP treatment is important. That two studies on therapeutic VIP treatment differed in whether they found an effect on colitis or not [37,38] further supports that inflammation parameters and treatment regimens are important factors for the outcome of VIP treatment and that such treatment would have to be carefully titrated to have beneficial effects. Furthermore, although the prophylactic treatment study found improved colitis at day 10 post infection, it can not be excluded from that study that later time points could have shown a worse outcome, or at least lack of improvement compared to control: as VIP has anti-inflammatory properties, which are important for clearance [40], this may impede pathogen clearance.

Even though VIP had beneficial effects on mitochondria and epithelial cells in the 5–10 day treatment regimen, there was no clear benefit on colitis or infection clearance overall. The only potential beneficial parameters detected were related to pathogen localization: fewer VIP treated mice tended to have systemic infection (2/12) compared to the non-treated infected mice (3/7), and the amount of *C*. *rodentium* in contact with epithelial cells tended to be lower in VIP treated mice compared to non-treated infected mice. That the improved health of the epithelial cells does not have overall beneficial effects on the infection may be due to that other factors fighting infection decrease simultaneously. Firstly, the infection induced mitochondrial damage and apoptosis is likely due to oxidative damage, since the mitochondria targeting antioxidant MitoQ, which scavenges peroxynitrite protects against these effects [14]. VIP reduced NO generation caused by *C*. *rodentium*, TNFα and IFNγ, both *in vivo* and *in vitro*. Although NO cause mitochondrial damage, several studies have shown a role for NO in mucosal defense against pathogens [59,60], and this may be the reason that VIP supplementation is not overall beneficial for treating infection. Secondly, mice lacking cytokines of Th1 and Th17 origin (IL-12, IL-22 and IL-17) are more vulnerable to infection [41–43], indicating that these also have roles in controlling *C*. *rodentium* infection. Furthermore, cellular components of the immune system (CD4^+^ T cells, neutrophils, B cells) are also important for pathogen clearance [41, 44–47]. The impact of VIP on these clearance inducing immune factors during *C*. *rodentium* infection is unknown. However, the anti-inflammatory action of VIP is exerted via several mechanisms, including inhibition of production of proinflammatory cytokines (i.e. TNFα and IFNγ) and increased production of anti-inflammatory cytokines [16–18]. Furthermore, enhanced production of IL-17 occurs in VIP knockout mice, [48] indicating that VIP may decrease IL-17 production in inflammatory disease conditions.

Using *in vivo*-like *in vitro* mucosal surfaces, we found that *C*. *rodentium* and TNFα, both individually and more severely in combination with IFNγ, decreased the levels and activity of the complex I and IV enzymes, mitochondrial phosphorylation capacity, transmembrane potential and ATP generation. That VIP is protective *in vitro*, suggests that the decreased levels of VIP occurring in the colonic epithelial cells during infection may lead to that the infection induced expression of TNFα and IFNγ inflict even greater damage than what might have occurred otherwise. Although our results indicate that VIP i.p. injection is not a promising treatment for infection, we can thus not exclude that a more local treatment, for example restoring VIP in the epithelial cells only, may be more functional.

In summary, we found that that mucosal VIP decrease during murine *C*. *rodentium* infection with a similar time dependency as measurements reflecting mitochondrial function and epithelial integrity. The loss of VIP is mainly cytokine driven, and VIP supplementation alleviates mitochondrial dysfunction and epithelial apoptosis caused by *C*. *rodentium* infection, TNFα and IFNγ both *in vivo* and *in vitro*. The protective action of VIP occurs by inhibition of damaging NO-generation caused by the infection, both indirectly via cytokines and directly via pathogens. However, VIP treatment failed to alleviate colitis although it tended to affect pathogen localization *in vivo*.
